# Ruminant self-medication against gastrointestinal nematodes: evidence, mechanism, and origins☆

**DOI:** 10.1051/parasite/2014032

**Published:** 2014-06-30

**Authors:** Juan J. Villalba, James Miller, Eugene D. Ungar, Serge Y. Landau, John Glendinning

**Affiliations:** 1 Department of Wildland Resources, Utah State University 5230 Old Main Hill Logan Utah 84322-5230 USA; 2 Department of Pathobiological Sciences, School of Veterinary Medicine, Louisiana State University 70803 Baton Rouge USA; 3 Department of Natural Resources, Institute of Plant Sciences, Agricultural Research Organization, the Volcani Center Bet Dagan 50250 Israel; 4 Department of Biology, Barnard College, Columbia University 3009 Broadway New York NY USA

**Keywords:** Diet selection, Foraging behavior, Condensed tannins, Learning

## Abstract

Gastrointestinal helminths challenge ruminants in ways that reduce their fitness. In turn, ruminants have evolved physiological and behavioral adaptations that counteract this challenge. Ruminants display anorexia and avoidance behaviors, which tend to reduce the incidence of parasitism. In addition, ruminants appear to learn to self-medicate against gastrointestinal parasites by increasing consumption of plant secondary compounds with antiparasitic actions. This selective feeding improves health and fitness. Here, we review the evidence for self-medication in ruminants, propose a hypothesis to explain self-medicative behaviors (based on post-ingestive consequences), and discuss mechanisms (e.g., enhanced neophilia, social transmission) that may underlie the ontogeny and spread of self-medicative behaviors in social groups. A better understanding of the mechanisms that underlie and trigger self-medication in parasitized animals will help scientists devise innovative and more sustainable management strategies for improving ruminant health and well-being.

## Abstract (fr)

Les helminthes gastro-intestinaux posent aux ruminants un défi en matière de fitness. À leur tour, les ruminants ont évolué des adaptations physiologiques et comportementales qui s’opposent à ce défi. Les ruminants présentent de l’anorexie et des comportements d’évitement, qui tendent à réduire l’incidence du parasitisme. En outre, les ruminants semblent apprendre à s’auto-médicamenter contre les parasites gastro-intestinaux en augmentant leur consommation des composés secondaires de plantes qui ont une action antiparasitaire. Cette alimentation sélective améliore santé et fitness. Dans ce travail, nous passons en revue les éléments de preuve de l’automédication chez les ruminants, proposons une hypothèse pour expliquer les comportements d’automédication (sur la base des conséquences post-ingestives), et discutons des mécanismes (par exemple, néophilie améliorées, transmission sociale) qui peuvent sous-tendre l’ontogenèse et la propagation des comportements d’automédication dans les groupes sociaux. Une meilleure compréhension des mécanismes qui sous-tendent et déclenchent l’automédication chez les animaux parasités aidera les scientifiques à élaborer des stratégies de gestion innovantes et plus durables pour améliorer la santé et le bien-être des ruminants.

## 1. Introduction

A herbivore’s existence is closely bound to that of parasites and pathogens [37]. Parasitism is a persistent challenge to their survival and reproduction [40–47], causing negative impacts on their health, daily activities [37], well-being [78], and nutritional state [13]. Collectively, all these effects lead to significant reductions in herbivore fitness [74]. To combat parasitic infections, herbivores generate immune responses that both disrupt parasite establishment and/or development [13, 19] and enhance their ability to cope and maintain productivity in response to a parasitic challenge [1]. In addition, ruminants have evolved behavioral means to suppress (or prevent) the negative impacts of parasitism. For instance, grazing ruminants minimize the chances of infection by avoiding areas where parasite larvae are most concentrated [19, 39, 40]. Horses grazing in highly stocked pastures exhibit “latrine behavior” (i.e., defecate in restricted areas), whereas their counterparts grazing in rangelands, where the parasite risk is lower, defecate randomly while grazing [55].

Another behavioral mechanism for minimizing parasite infection is to self-select foods containing compounds that help treat or control parasite infections. This field of study is referred to as zoopharmacognosy or self-medication [33, 36]. It is based on the postulate that animals have evolved behavioral and physiological mechanisms to treat or control disease and/or its symptoms, leading to an improvement in health and, as a consequence, an enhancement in fitness [37]. In a seminal study, Huffman and Seifu [35] observed that wild chimpanzees suffering from parasite-related diseases consumed the bitter pith of the plant *Vernonia amygdalina* which contains sesquiterpene lactones and steroid glucosides – compounds with antiparasitic activity at the doses consumed by the animals [49]. Since these pioneering results, additional evidence pointing to the use of plant secondary compounds to recover or maintain health has been reported not only for chimps and other mammals [30], but also for insects [74] and birds [12]. The study of self-medication seeks to understand (a) how animals respond behaviorally to challenges that potentially affect their health, and (b) how these behaviors are maintained within a population [37].

In this paper we present evidence of self-medication in ruminant animals and propose mechanisms by which self-medicative behaviors are acquired. Our primary focus is self-medication in the context of ruminant animals infected with gastrointestinal endoparasites (primarily nematodes), and our aim is to stimulate further research aimed at understanding the underlying mechanisms and ontogeny of self-medicative behaviors. A deeper understanding of the mechanisms and triggers of self-medication by parasitized animals should pave the way to innovative management strategies to improve animal health and welfare in a sustainable way with reduced human interventions.

## 2. Evidence of self-medication in parasitized ruminants

Self-medicative behaviors, whereby plants containing natural anthelmintics are selectively ingested by ruminants, can be classified into two categories: prophylactic and therapeutic [32]. Goat kids from the Mamber breed, which generally exhibit low propensity to consume the antiparasitic shrub *Pistacia lentiscus* [56], increased their preference for this plant following infection with 10,000 L3 larvae of mixed gastrointestinal nematodes, suggesting therapeutic self-medication [2]. In contrast, goat kids from the Damascus breed typically ingest high amounts of the aforementioned plant irrespective of infection [25], thus exhibiting prophylactic self-medication. Prophylactic ingestion of medicinal plants encompasses behaviors that are likely rooted in fixed action patterns and genetic adaptations which are not necessarily linked to the current physiological state of an animal. While therapeutic self-medication emerges from a learning process involving the interaction between the orosensorial characteristics of foods and their post-ingestive medicinal effects (i.e., a feedback mechanism), prophylactic self-medication can be explained through a preventive “feedforward” mechanism [89]. The present review will focus on the emergence of functional behavioral responses to the challenges imposed by endoparasites with an emphasis on therapeutic self-medication.

Surveys and field observations, as well as controlled studies, indicate that parasitized ruminants exhibit self-medication. For instance, surveys have revealed that parasitized goats self-select unpalatable plants with anthelmintic properties [30] and ingest higher percentages of heather containing tannin (an anthelmintic plant secondary compound) than anthelmintic-treated goats [64]. Likewise, sheep infected with adult populations of *Haemonchus contortus* eat more of the tannin-rich plant *Lysiloma latisiliquum* (Tzalam) than non-infected animals [59].

In a controlled experiment, lambs experiencing natural gastrointestinal helminth burdens ate more of a tannin-rich supplement than non-parasitized animals, even when the supplement was of very low nutritional value. In contrast, non-parasitized lambs consumed more of the supplement without tannins than parasitized lambs [58]. Likewise, lambs with natural gastrointestinal parasitic burdens and non-parasitized lambs were offered a choice of alfalfa (*Medicago sativa*) and a mix containing 90% alfalfa and 10% quebracho tannin. Parasitized lambs showed a greater preference for the tannin-containing food than non-parasitized animals; these differences disappeared when parasite loads were eliminated by chemotherapy [86].

Previous experience and learning play a role in self-medication. For instance, when lambs were infected with 10,000 L3 (infective larvae) of *H. contortus*, intake of and preference for a tannin-rich feed was high for lambs that had previously experienced the beneficial effects of condensed tannins while parasitized; intake was comparatively low in lambs lacking this experience. When parasitic infections were terminated by chemotherapy, differences between groups disappeared [43]. This suggests that individual experience with the medicinal effects of tannins enhances intake of and preference for tannins during subsequent parasite infections.

## 3. A functional explanation for self-medication in ruminants

The senses of smell, taste, mouthfeel, and sight enable animals to discriminate among foods. Post-ingestive feedback calibrates these sensory experiences – like or dislike – in accord with a food’s positive or negative post-oral effects [69]. Such dynamic integration of chemosensory and post-ingestive signals gives ruminants the ability to modify diet selection as a function of the consequences of food experienced throughout their lifetimes [71].

Nutritional state and dietary experience in ruminants can modify ingestive responses to foods. Lambs fed diets low in energy and protein prefer flavored foods previously paired with intra-ruminal infusions of energy (starch, propionate, acetate) or nitrogen (urea, casein, gluten) [82–85]. From a functional standpoint, ingesting medicine is not so different from acquiring nutrients while foraging. If herbivores can learn to prefer nutritious foods, they may also learn to prefer medicinally beneficial foods [43].

**Figure 1.**
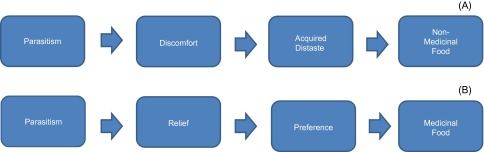
(A) Gastrointestinal parasites cause pain and discomfort. Non-medicinal foods associated with such sensations may lead to an acquired distaste. (B) In contrast, medicinal antiparasitic foods that promote relief can lead to a preference, i.e., self-medication.

The most significant effect of gastrointestinal parasites is a depression in food intake [76], which is partially attributed to pain and discomfort associated with the infection as well as to hormonal changes from disrupted gastrointestinal function [77]. Helminths damage gut tissue and impair mucoprotein secretion [8, 75]. For instance, the larvae of *Trichostrongylus* in the small intestine may cause severe damage to the intestinal mucous membrane, and the L_4_ larval stage and adults of *Haemonchus* are blood feeders that cause anemia and disrupt mucous membranes in the abomasum [15]. In addition, it has been suggested that the cytokine release that accompanies infection reduces food intake [57]. All these effects can cause discomfort and gastrointestinal distress, which decreases food palatability and increases the formation of acquired distastes [67] (Fig. 1). Thus, it is expected that parasitized animals will readily acquire a distaste for a non-medicinal diet. Consistent with this expectation, parasitized sheep exhibit a low preference for the non-medicinal legume cicer milkvetch relative to a baseline period before infection [89]. On the other hand, animals should be able to learn about the medicinal benefits of a food and acquire a preference for that food when its ingestion is associated with relief from illness or discomfort [88]. In support of this possibility, reductions in gastrointestinal distress have been found to increase food palatability and condition preferences in rats [67]. Herbivores may also be able to develop a conditioned preference for foods associated with recovery from gastrointestinal distress [88] (Fig. 1). This may explain why parasitized lambs exhibit a higher preference for the tannin-containing legume sainfoin than non-parasitized lambs [89].

It has been proposed that post-ingestive consequences (e.g., illness) from a food must occur shortly after feeding in order for ruminants to condition an aversion to that food [91]. However, more recent research has shown that ruminants can condition aversions to toxic foods following delays of up to 8 h between food ingestion and consequences [10], and can condition preferences for calorically rich foods following delays of up to 1 h [86]. Relief from parasitism after medicinal plant ingestion may commence within 1–8 h [61], suggesting that this time delay will not prevent herbivores from learning about the positive post-ingestive effects of medicinal plants on parasitic burdens.

Nevertheless, some goat breeds exhibit high propensity to consume an anthelmintic tannin-rich food even when they are naïve to it and even when they do not experience a worm load concomitantly to ingesting tannins [2]. This observation raises the possibility that whereas some ruminants must condition preferences for foods with anthelmintic properties, others possess innate preferences for them.

## 4. The triggers for self-medication in ruminants

From the previous analysis, we can infer that ruminants need to ingest doses of an antiparasitic medicine (i.e., a plant secondary compound) in order to experience its benefits and thus learn to self-medicate. Indeed, Mediterranean goats in tannin-rich environments will typically ingest 1 g/kg BW of PEG-binding tannins [25], which is the dose needed to suppress fecal excretion of nematode eggs almost entirely [56]. However, many antiparasitic medicines found in nature are secondary compounds, which evolved to deter herbivory by inducing negative post-ingestive effects [66]. In addition, many antiparasitic secondary compounds have a bitter taste. Garcia and Hankins [21] maintained that mammals should avoid anything that tastes unpalatable, particularly if it is bitter. This rule is supported by the observations that virtually all naturally occurring poisons taste bitter to humans [28], and most chemicals that taste bitter to humans also elicit an aversive response in other mammals [27]. In addition, many plant secondary compounds deter herbivory by eliciting burning, sour and astringent oral sensations. Some others also produce unpleasant odors or irritating sensations in the nose [27, 28]. Thus, secondary compounds provide unpleasant oral and nasal sensations, which deter feeding by herbivores.

What mechanisms allow parasitized ruminants to ingest therapeutic doses of potentially toxic chemicals that also induce unpleasant taste and odor sensations? It is known that ruminants change their behavior in response to parasitism. We will review some of these changes and propose some others in an attempt to answer this question.

### 4.1 Parasitism and changes in feeding behavior

Several behavioral changes caused by endoparasites are rooted in the induction of anorexia. Anorexia is a common symptom of parasitic infectious diseases, but one that has been qualified as paradoxical because parasites typically impose increased metabolic and nutritional demands on the host [50, 54]. Anorexia is manifested in parasitized ruminants as shorter and fewer feeding bouts relative to non-infected individuals [16–18]. In addition, the extent, duration and rate of recovery of pathogen-induced anorexia are influenced by the type of food available. Parasitized individuals consuming a nutritious food are anorexic for a shorter period of time than individuals exposed to a food of lower nutritional quality [54].

Rather than being a paradoxical response, anorexia may in fact represent a behavioral adaptation [6]. For instance, it has been hypothesized that anorexia allows the host to become more selective in its diet, and thus choose foods that either minimize the risk of infection or augment the intake of antiparasitic compounds [53]. For instance, when parasitized herbivores were offered a choice between non-contaminated and feces-contaminated pastures, they avoided the latter pastures [14, 39]. Avoidance of feces has been reported in non-parasitized animals, but this behavior appears to be more pronounced in parasitized animals [39]. The avoidance of feces by parasitized animals occurs even when feces-contaminated fields offer higher nutrient rewards [40]. When infected animals were forced to consume contaminated pastures, they grazed further from the soil surface than non-parasitized animals, thereby minimizing the risk of parasite intake [40].

There is also evidence for the hypothesis that parasitized animals become more selective. When sheep infected with larvae of the intestinal nematode *Trichostrongylus colubriformis* were given a choice between two feeds that differed in protein content, they exhibited unusually high preferences for the high-protein feed, likely to meet the increased protein requirements due to parasitism [51, 52]. However, an increase in protein selection was not observed in dairy heifers sub-clinically infected with *Cooperia oncophora* and *Ostertagia ostertagi* [18].

Endoparasites also affect other aspects of behavior. It has been shown that parasitized animals take fewer steps, lie longer and change posture less frequently than non-parasitized animals [78]. This reduced activity may be a consequence of lethargy likely aimed at conserving energy [31], or as another parasite-avoidance strategy [53].

Even when parasitism induces anorexia and lethargy, enhanced preferences for medicinal plants may nevertheless lead to an increased dose of antiparasitic agents consumed with the diet, despite the fact that overall food intake may decline. More work is needed to explore this possibility.

### 4.2 Neophilia

**Figure 2.**
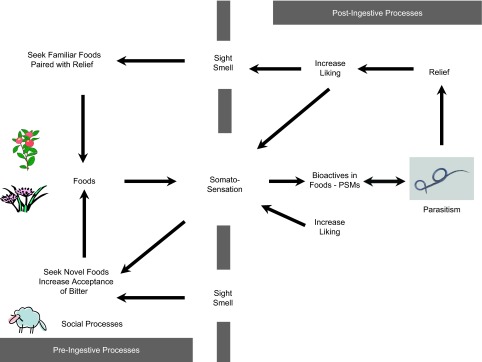
A conceptual representation of how pre- and post-ingestive events control the manifestation of self-medicative behavior in mammalian herbivores. Self-medication emerges from enhanced neophilia and increased acceptance of certain somatosensations (e.g., taste dimensions, tactile properties) triggered by parasitism. These increases in neophilia and acceptability, together with social learning, should “prime” animals to ingest therapeutic doses of medicinal secondary compounds (pre-ingestive processes). Subsequently, associative learning (i.e., associations between orosensorial properties of a medicinal food and relief experienced after ingesting that food) will maintain and/or reinforce self-medicative behaviors. Thus, a chain of events starting with food acceptability and social learning followed by post-ingestive processes may contribute to the emergence of self-medication in mammalian herbivores.

In addition to anorexia and avoidance behavior, parasitism may increase the attractiveness of novel foods and orosensory stimuli (i.e., lead to enhanced neophilia), and thereby increase the likelihood that therapeutic doses of prophylactic plant secondary compounds will be consumed (Fig. 2).

When physiological requirements are met and the animal is in a homeostatic state – such as when balanced rations are available *ad libitum* – ruminants eat only small amounts of novel foods. This neophobic behavior likely evolved to decrease the likelihood of consuming harmful foods. However, departure from homeostasis (e.g., when there is an inadequate or unbalanced supply of nutrients) may cause neophilia [70]. Likewise, birds on a positive energy budget are averse to risk, but prone to it when on a negative energy budget [11]. This is because individuals that have acquired abundant reserves of energy have more potential fitness to lose from taking risks (e.g., selecting novel foods, exploring new places) than individuals which have not [9]. Likewise, sick individuals may have less potential fitness to lose from taking risks (e.g., by selecting novel foods or foods containing novel plant secondary compounds) than healthy ones. Thus, one would expect that when homeostasis is disturbed by parasite infection (which imposes metabolic constraints), animals should become more neophilic. This prediction has been explored recently in our laboratories: parasitized goats increased their intake of tannin-rich *P. lentiscus* relative to non-parasitized counterparts if they had been conditioned before infection to hay or to another browse species, but not if they had been conditioned to *P. lentiscus* [2]. Also, we offered parasitized (*H. contortus*) and non-parasitized lambs a novel food (beet pulp) with increasing concentrations of condensed tannins. Lambs had a trinary choice of beet pulp containing 0%, 5%, and 10% quebracho tannins. First, consumption of the novel beet pulp with 0% tannins was greater in parasitized than non-parasitized animals. Second, during the initial 2 d of exposure, parasitized animals preferred the beet pulp without condensed tannins, but by day 6 they ingested a greater proportion of beet pulp adulterated with condensed tannins than non-parasitized lambs (Egea, Miller, Hall, and Villalba, unpublished results). Thus, parasitized lambs showed initially a greater degree of acceptance of the novel food with a taste salience presumably similar to the (familiar) basal diet of alfalfa pellets. For a consumer, taste salience depends on novelty of the taste not on concentration [46], and the taste of tannins was certainly more novel than that of beet pulp. Since beet pulp did not provide a medicinal effect, it is possible that on subsequent days parasitized lambs displayed an increased acceptance of the “more novel” tannin-containing foods, which provided a medicinal effect to the consumers. Alternatively, when rodents repeatedly sample tannins, they induce production of salivary proline-rich proteins (PRPs) which bind to the tannins and render them more palatable [26]. If the same induction process for tannin-binding salivary proteins occurs in lambs, as has been suggested in lambs exposed to tannin-rich forage [81], then this could explain the gradual increase in intake of the tannin-treated diet by parasitized animals, which repeatedly sampled such a diet.

Increased acceptance of certain taste sensations may substantially benefit herbivores. For instance, if herbivores could tolerate the taste of bitter foods, then they could substantially expand their range of dietary options (Fig. 2). They could also increase their intake of therapeutic secondary compounds, which typically taste bitter [27, 37]. Repeated sampling of the bitter-tasting anti-malarial agent chloroquine by malaria-infected mice resulted in significant reductions in parasitemia and risk of mortality [90]. Thus, there is evidence in mouse models that acceptance of bitter taste has the potential to enhance fitness when consumers are challenged by parasitism. This response can be explained by a preventive “feedforward” mechanism or prophylactic self-medication [90]. Feedforward mechanisms can also explain why there is a fine line between medicine and food in both primates and indigenous peoples in different parts of the world. In mountain gorillas, for instance, 30% of their daily herbaceous diet contains plant secondary compounds with antibacterial properties. Of the 172 plant species typically consumed by Mahale chimpanzees, 22% are used to treat gastrointestinal-related illnesses in humans. In addition, 89% of the species used to treat symptoms of malaria among the Hausa of Nigeria are also used in a dietary context [33]. Antioxidants such as flavonoids reduce the negative impacts of free radicals in the body and birds preferentially select flavonoids in their diets, which leads to lower oxidative stress and enhanced immunity, presumably through prophylactic self-medication [12]. Would feedforward mechanisms eventually prime animals to consume therapeutic doses of anthelmintics and thereby enhance their ability to learn about the medicinal post-ingestive effects of foods? The answer to this question may be affirmative, but more research is needed in this regard; we still do not know whether parasitized ruminants feed preferentially on anything that tastes bitter, unpleasant, or novel. If parasite infections cause animals to increase intake of unpleasant-tasting plant tissues, then what is the mechanism underlying this phenomenon? Does the food remain unpalatable, but the animal increases its tolerance for the orosensory properties of the food? Alternatively, does parasite infection make animals less sensitive to unpleasant-tasting plant tissues? In caterpillars, infection by parasites enhances acceptance of the taste of some antiparasitic pyrrolizidine alkaloids, which has a positive impact on fitness [7].

### 4.3 Social models

Social models play an important role in diet selection and preferences of young animals [20]. For instance, the degree of affinity of lambs with their social models affects their acceptability of novel foods [79]. Socializing enhances learning efficiency because each animal no longer has to discover everything through trial and error (e.g., after experiencing the positive or negative post-ingestive consequences of a novel food). Pioneering animals in a social group may learn about the beneficial effects of specific foods or combinations of foods. Once this pioneer develops a preference for a food, the preference may then spread through the group, becoming part of the foraging behavior of females [36], who can then transmit those behaviors to their offspring. Cultural transmission and maintenance of leaf swallowing (a self-medicative behavior directed to combat gastrointestinal endoparasites) within a group and subsequent associative learning by the individual of the positive consequences of this behavior has been proposed to be the pivotal link between the propensity for consuming leaves and its maintenance as a self-medicative behavior by great apes in the wild [38]. Such transmission of information across generations occurs in livestock. For instance, when offered a choice, lambs tend to show the same food preferences and aversions as their mothers [62, 63]. Mothers also appear to play a critical role in “teaching” naïve lambs self-medicative behaviors [72]. Mediterranean goats consume tannin-rich *Pistacia lentiscus* even at times of plentiful green herbage, and educate their kids to consume the plant in high (Damascus goats) or moderate amounts (Mamber goats) [24]. This could be the basis of self-medication behavior, as ingesting *P. lentiscus* foliage decreases the incidence of nematode eggs in feces to almost zero [56] and impairs development of gastrointestinal larvae [4]. By educating their kids to consume *P. lentiscus* moderately [24], Mamber does prepare the ground for therapeutically increased intake of this shrub in case of infection. In contrast, Damascus goats encourage high propensity to consume the shrub, i.e., educate their kids to self-medicate in a prophylactic way.

There is a trade-off between social information and competition: grazing in a group is more efficient, and animals spend less time evaluating which foods to eat and which foods to avoid. Grazing alone involves less competition with peers [73], but grazing in a group increases the odds of being infected by parasites.

Interestingly, in a Mediterranean rangeland dominated by tannin-rich *Quercus coccifera*, the most dominant goats consumed more shrubs than lower-ranked counterparts [5]. In other words, aggressiveness was not directed at consuming the most nutritious diet, but instead, at consuming a diet that protects consumers from parasites. A survey carried out in the Cazorla Natural Park of Spain [22] showed that wild goats (*Capra pyrenaica*) consumed a diet consisting of 41% browse and 59% herbaceous vegetation, whereas domestic goats (*Capra hircus*) selected a diet with 81% browse and 19% herbaceous vegetation; and the diets of wild sheep (*Ovis musimon*) contained 80% herbaceous species, compared with 48% for domestic sheep (*Ovis aries*), which also included 25% of dwarf shrubs in their diets. Collectively, the information presented suggests that it is likely that social models in domesticated ruminants “prime” the individual to increase consumption of an otherwise typically avoided secondary compound-containing food (Fig. 2).

## 5. Integration of pre- and post-ingestive events on self-medication

If a plant’s tissues contain a secondary compound that has anti-parasitic properties but does not confer unpalatability, then the likelihood of parasitized herbivores consuming the plant (and perhaps benefitting from its medicinal effects) would be high. In contrast, if another plant’s tissues contain a secondary compound that has antiparasitic properties but makes the plant unpalatable, then the likelihood of parasitized herbivores consuming this plant (and perhaps benefitting from its medicinal effects) would be comparatively low. Low or non-therapeutic doses of medicines should prevent animals from experiencing the potential medicinal benefits of such compounds, and thereby prevent the emergence of self-medicative behavior. However, if sick animals “cross the rejection threshold” and experience the benefits of consuming the medicine, they may continue ingesting the medicinal compounds and the behavior may persist in time with the potential to be transmitted within a social group. In this case the therapeutic benefits may outweigh the toxic effects, particularly for secondary compounds such as tannins, which have relatively low toxicity [64]. Thus, if the medicinal benefits of pharmaceutically active compounds overrule the toxic effects and increase fitness in parasitized individuals, then it is expected that consumers will “cross the rejection threshold” in such a scenario (e.g., [58, 74]). In some instances, herbivores may need to experience the post-ingestive effects of an unpalatable medicinal food in a conditioning session where no food alternatives are available, so this intervention would help the animal “cross the rejection threshold” imposed by the foods’ orosensorial properties.

In the wild, mammalian herbivores are capable of regulating their intake of secondary compounds. It has been proposed that this regulation occurs through post-oral mechanisms that influence the size of a meal and the inter-meal interval [80]. In brushland, free-ranging goats consume diets with 3%–3.5% of condensed tannins throughout the year, in spite of the extreme variability of browse and herbage on offer [45]. These mechanisms involve sensing secondary compounds in the gut and blood, as well as conditioned food aversions [29, 80]. The idea that ingestion of secondary compounds is a regulated process, which seeks to keep ingested secondary compounds at a subtoxic level, raises the possibility that these compounds may have a prophylactic action against intestinal parasites at the doses consumed by infected herbivores. Self-medication is also a regulated process (instead of an all-or-none process) in which animals incorporate certain sub-toxic doses of plant secondary compounds into their diets and sometimes for only some days during infection, instead of manifesting strong preferences for secondary compound-containing foods [35, 44].

## 6. Conclusions and future directions

Over the past 40 years, we have gained insight into how nutritional deficiencies alter feeding preferences in ruminants. We are just starting to understand how parasitic infections and illness alter the onset of self-selection of medicines in these animals. Emerging evidence suggests ruminants are able to self-medicate by ingesting plant tissues that contain pharmaceutically active compounds. However, less clear are the mechanisms by which self-medicative behaviors are initiated and evolve in ruminants. Even if self-medication can be attributed to a behavioral tradition, this leaves open questions about how the behavior was individually acquired in the first place and how individuals become predisposed to ingest medicinal plants [36]. A better understanding about the mechanisms that are involved in the learning process of self-medication will help managers devise innovative strategies that enhance the use of plants containing antiparasitic compounds.

Disease caused by gastrointestinal nematodes is one of the most important health constraints affecting productivity in small ruminants [42]. There is an increased interest in finding alternative treatments for parasite control due to the fact that pathogens are rapidly developing resistance to existing drugs [41]. For farm animals in particular, there is a clear desire to create systems of production that rely less heavily on chemotherapy [3]. Self-medication represents a sustainable and targeted strategy of parasite control [34] since animals can potentially select medicinal plants as a function of need. Self-medication also enhances the nutrition and welfare of ruminant animals as they do not need to be forced to consume a monoculture of a certain bioactive-containing plant; they can self-select the medicinal plant while they still have an array of nutritive alternatives available.

Despite the aforementioned benefits, more research is needed to understand the mechanisms that trigger and sustain the utilization of antiparasitic plants and supplements better. For instance, we need more knowledge on how ruminants experience malaise during a parasitic infection and how they experience relief after consuming an antiparasitic food (Fig. 1). How do animals identify medicinally active plants? When they get sick, do they simply feed preferentially on anything that tastes/smells unpleasant? Do they learn from other animals what to eat? Do they use a trial-and-error system? For instance, if parasitic infections cause animals to increase intake of unpleasant-tasting/smelling plant tissues, then what is the mechanism underlying this phenomenon? Does the food remain unpalatable, but the animal tolerates its bad taste/smell and eats it anyway? Alternatively, does the orosensation of a medicinal food become more acceptable during sickness?

It is known that some by-products of infection such as lipopolysaccharides, peptidoglycans, and microbial nucleic acids stimulate the production of proinflammatory cytokines, which reach the central nervous system and serve as endogenous mediators of anorexia [57]. Damage of gut tissue may lead to local inflammation [8, 75]. Gastrointestinal parasitism causes damage to the gastrointestinal mucosa, which results in increased plasma leakage and losses of endogenous protein to the lumen [68]. Endoparasites may also disrupt absorption and retention of nitrogen, minerals, and vitamins [23, 48]. Thus, central and peripheral mechanisms may trigger discomfort in the host. In turn, substances that block cytokine synthesis/action and/or reduce inflammatory responses or pain may lead to an alleviation of the discomfort experienced by parasitism and thus enhance liking for the food associated with this sensation.

More research is also needed for elucidating the mechanisms that trigger acceptance of antiparasitic substances by the host. It is likely that parasitism triggers the upregulation of specific taste receptors which in turn increase the acceptance of certain taste dimensions (i.e., bitter) and thus the increased intake of antiparasitic foods. In caterpillars, infection by parasites alters the taste response for specific medicinal plant secondary compounds (pyrrolizidine alkaloids) which encourages secondary compound ingestion, providing a biochemical defense [7]. Novel genes for bitter taste reception are being identified in ruminants [19] and this knowledge can contribute to designing experiments in parasitized and non-parasitized animals that lead to a better understanding of the triggers of self-medicative behavior in ruminants. Ingestion of bacterial lipopolysaccharides alters sweet taste function in mice [92]. These lipopolysaccharides seem to mediate the production of proinflammatory cytokines that downregulate sweet taste receptors genes in taste buds [92]. Thus, it is likely that some by-products of infection influence taste sensation in infected animals. Finally, more research is needed to establish the link between social transmission of information regarding diet selection and the ability of ruminants to self-medicate.
